# The Patient-Specific Combined Target Zone for Morpho-Functional Planning of Total Hip Arthroplasty

**DOI:** 10.3390/jpm11080817

**Published:** 2021-08-21

**Authors:** Juliana Habor, Maximilian C. M. Fischer, Kunihiko Tokunaga, Masashi Okamoto, Klaus Radermacher

**Affiliations:** 1 Chair of Medical Engineering, Helmholtz-Institute for Biomedical Engineering, RWTH Aachen University, 52074 Aachen, Germany; juliana.habor@rwth-aachen.de (J.H.); Maximilian.Fischer@rwth-aachen.de (M.C.M.F.); 2 Niigata Hip Joint Center, Kameda Daiichi Hospital, Niigata City 950-0165, Japan; ktokunagajp@yahoo.co.jp; 3 Department of Radiology, Kameda Daiichi Hospital, Niigata City 950-0165, Japan; rock.annonymous@gmail.com

**Keywords:** total hip arthroplasty, preoperative planning, patient-specific THA, target zone, safe zone, leg length discrepancy, range of motion, edge loading

## Abstract

**Background** Relevant criteria for total hip arthroplasty (THA) planning have been introduced in the literature which include the hip range of motion, bony coverage, anterior cup overhang, leg length discrepancy, edge loading risk, and wear. The optimal implant design and alignment depends on the patient’s anatomy and patient-specific functional parameters such as the pelvic tilt. The approaches proposed in literature often consider one or more criteria for THA planning. but to the best of our knowledge none of them follow an integrated approach including all criteria for the definition of a patient-specific combined target zone (PSCTZ). **Questions/purposes** (1) How can we calculate suitable THA implant and implantation parameters for a specific patient considering all relevant criteria? (2) Are the resulting target zones in the range of conventional safe zones? (3) Do patients who fulfil these combined criteria have a better outcome score? **Methods** A method is presented that calculates individual target zones based on the morphology, range of motion and load acting on the hip joint and merges them into the PSCTZ. In a retrospective analysis of 198 THA patients, it was calculated whether the patients were inside or outside the Lewinnek safe zone, Dorr combined anteversion range and PSCTZ. The postoperative Harris Hip Scores (HHS) between insiders and outsiders were compared. **Results** 11 patients were inside the PSCTZ. Patients inside and outside the PSCTZ showed no significant difference in the HHS. However, a significant higher HHS was observed for the insiders of two of the three sub-target zones incorporated in the PSCTZ. By combining the sub-target zones in the PSCTZ, all PSCTZ insiders except one had an HHS higher than 90. **Conclusions** The results might suggest that, for a prosthesis implanted in the PSCTZ a low outcome score of the patient is less likely than using the conventional safe zones by Lewinnek and Dorr. For future studies, a larger cohort of patients inside the PSCTZ is needed which can only be achieved if the cases are planned prospectively with the method introduced in this paper. **Clinical Relevance** The method presented in this paper could help the surgeon combining multiple different criteria during THA planning and find the suitable implant design and alignment for a specific patient.

## 1. Introduction

Major complications of total hip arthroplasty (THA) and reasons for revisions are infections, dislocations, wear and loosening [1,2,3]. Different studies found dislocation rates of 0.2% to 10% [4]. When considering 1% and over one million THA surgeries per year worldwide [4], the absolute numbers of dislocations would be over 10,000 cases per year. The number of young and active patients is increasing [5]. The revision rate for patients younger than fifty is higher than in older patients [6]. This indicates that more active lifestyle puts higher demands on the prosthesis [4]. The complications could be addressed by a proper choice of the implant components (size and shape, or implant parameters) and their alignment (position and orientation, or implantation parameters) within the patient’s bony structures. Various methods for finding suitable or optimal parameters have been introduced in the literature. Often, the term safe zone describes cup orientations with a low risk for dislocation. In this paper, a more general term, namely target zone, is used to describe a comprehensive set of suitable implant and implantation parameters (and not only the cup orientation).

There are studies using statistical analysis to find a correlation between the clinical outcome and the implant and implantation parameters. Lewinnek suggested aligning the cup with an inclination of 40° ± 10° and anteversion of 15° ± 10° (the so-called Lewinnek safe zone) in order to reduce the dislocation risk [7]. However, studies showed that the majority of dislocated or revised hips had cup orientations within the safe zone [8,9,10]. Other safe zones [11,12,13,14,15,16,17,18,19,20] and rules for combined anteversion of the cup and stem [16,17,21,22,23] were suggested. Dorr et al., for instance, found a safe range for combined anteversion of 25° to 50° [23]. However, these safe zones and ranges are not consistent with each other.

Further publications introduced methods for deriving optimal parameters by considering certain criteria, such as the range of motion (ROM) and prosthetic impingement [24,25,26,27,28,29,30,31,32], bony impingement [33,34,35,36,37], bony cup coverage [38,39,40], leg length discrepancy, wear rate and edge loading risk [31,32,41,42,43,44]. Dislocation is either caused by a levering-out motion due to impingement, or sliding-out motion when the resulting hip force is directed outside of the cup [31]. The hip force affects the wear rate and edge loading which influences the longevity of the implant.

The methods in literature often include one or more criteria but to the best of our knowledge none considers all criteria at once. Consequently, in many cases only a few relevant criteria are evaluated quantitatively whereas others are considered only implicitly or neglected in the daily clinical routine of the patient-specific surgical planning process. Often, a compromise between conflicting objectives has to be found. For instance, positioning the cup for maximal ROM might reduce the bony cup coverage. The cup and stem positioning, the caput–collum–diaphyseal (CCD) angle and the neck length have an influence on the leg length discrepancy but also on the hip force.

The pelvic tilt which has a high variability among different patients and between different postures (supine, standing, sitting) has a direct influence on the functional cup orientation [45,46,47]. Algorithms for calculating the changed cup orientation due to the pelvic tilt have been introduced [48,49,50,51,52]. Some THA planning methods include the pelvic tilt during calculation of edge loading [41] or during ROM analysis [29].

The aim of this study is to investigate the following questions:

1. How can we calculate suitable THA implant and implantation parameters for a specific patient considering the most relevant criteria?
2. Are the resulting target zones inside conventional safe zones?
3. Do patients with implantations fulfilling these combined criteria have a better outcome score?

## 2. Material and Methods

### 2.1. Patient-Specific Target Zone Calculation 

**Figure 1:**
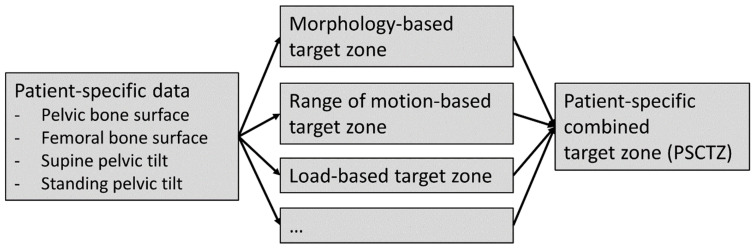
Overview of the concept for patient-specific combined target zone calculation.

We developed a method for calculating the patient-specific combined target zone (PSCTZ) incorporating a more comprehensive set of relevant criteria from (currently) three different target zones addressing different objectives of an optimal implant and implantation planning (Figure 1). Single target zones are calculated based on criteria related to the morphology, ROM, and load situation which are then merged into the PSCTZ. Other criteria can be added in a modular fashion. Hence, the PSCTZ is the overlap of all single target zones.

Patient-specific morphological and functional data including surface models of the pelvis and femur and the pelvic tilt of functional positions for daily living are needed to calculate each target zone. The surface models can be reconstructed from CT data as in most CT-based planning and navigation systems [53,54,55]. The pelvic tilt can be derived, for instance, from lateral radiographs [56], EOS imaging combined with CT [57], inclinometers [52] or navigated ultrasound [58,59].

The considered implant parameters are cup size, head/neck ratio, CCD angle and neck length. The considered implantation parameters are cup inclination, cup anteversion, 3D cup position, 3D stem position, stem ante-torsion, stem adduction, and stem flexion. These relate to the pelvic or femoral bone coordinate system. In the current study, the pelvic coordinate system is based on the anterior pelvic plane (APP). The center of rotation is the origin. For the pelvis in standing position, the APP is tilted relative to the frontal plane according to the patient-specific standing tilt, and the line connecting the left and right anterior superior iliac spine is parallel to the horizontal axis. The femoral coordinate system is based on the table top position [60] and the mechanical axis, with the center of rotation being the origin. The femur is in neutral position if the mechanical axis is parallel to the vertical axis and the line connecting the posterior condyles is parallel to the frontal plane.

The implant design and alignment influence the relative alignment of the pelvis and femur. The cup position defines the center of rotation. The femoral alignment depends on the stem position, stem orientation and the CCD angle, since these parameters change the head center position. Hence, a transformation is applied to the femur in order to realign the mechanical axis to the vertical axis.

#### 2.1.1. Morphology-Based Target Zone

**Figure 2:**
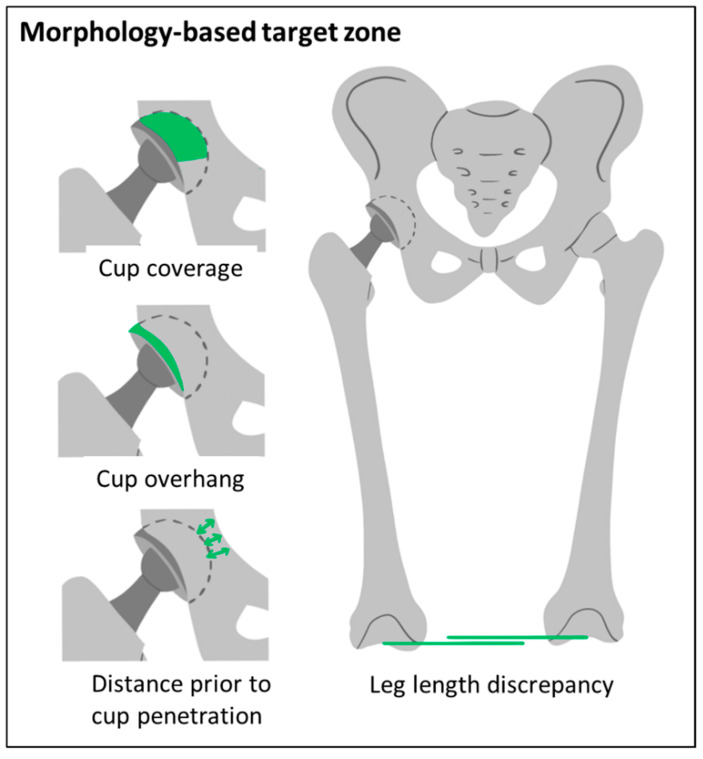
Criteria considered for the morphology-based target zone.

The criteria considered in the morphology-based target zones are related to the bony anatomy of the patient. The cup coverage, anterior cup overhang, distance prior to cup penetration, and the leg length discrepancy are calculated (Figure 2).

The cup is modelled as hemisphere. The overlapping area between the hemisphere and the acetabulum is determined for calculation of the percentage of coverage, similar to a method proposed by Ueno et al. [39]. The surface of the cup counts as covered if it overlaps by more than 0.5 mm. Then, it is determined which part of the uncovered area of the cup is overhanging and the maximal anterior cup overhang is calculated. Furthermore, the shortest distance from the outer shell of the cup and the medial surface of the pelvic bone is calculated, defining the distance prior to cup penetration. Lastly, the leg length discrepancy is determined by comparing the height of the intercondylar notch on both sides. The bones are neutrally aligned for all calculations.

All implant and implantation parameters that satisfy the following criteria are considered as within the morphology-based target zone:

- A bony coverage of at least 65%.
- An anterior cup overhang of less than 12 mm.
- A distance prior to cup penetration of at least 1 mm,
- A maximal leg length discrepancy of ±8 mm.

A minimum cup coverage of 60% measured in anterior-posterior radiographs [40] or 61.2% measured on the upper portion of a 3D cup model [39] were recommended in recent studies. A more conservative threshold of 65% was chosen here. A study showed that patients with iliopsoas impingement on the acetabular cup which might induce pain had anterior cup overhang of more than 12 mm measured in CT data [61]. The distance prior to penetration was considered in automated planning methods [62,63]. A limit of 1 mm was chosen in order to prevent penetration. A leg length discrepancy of 10 mm was stated as a critical threshold [64]. A more conservative 8 mm was chosen here. These thresholds can be adjusted to the individual patient or other standard values.

#### 2.1.2. ROM-Based Target Zone

**Figure 3:**
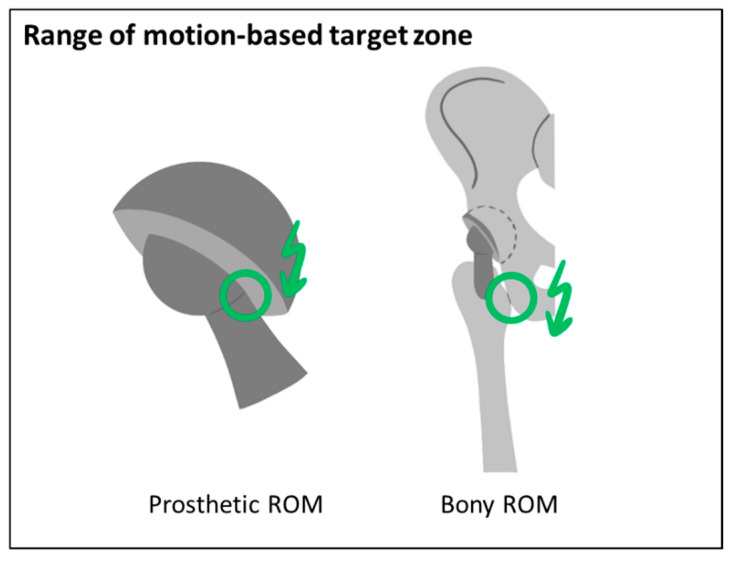
Criteria considered for the ROM-based target zone.

The criteria considered in the ROM-based target zones are related to the prosthetic and bony impingement risk while performing a target ROM (Figure 3). The target ROM can be defined based on literature data [27,30,65,66] and might be adapted based on patient-specific characteristics and requirements.

**Figure 4:**
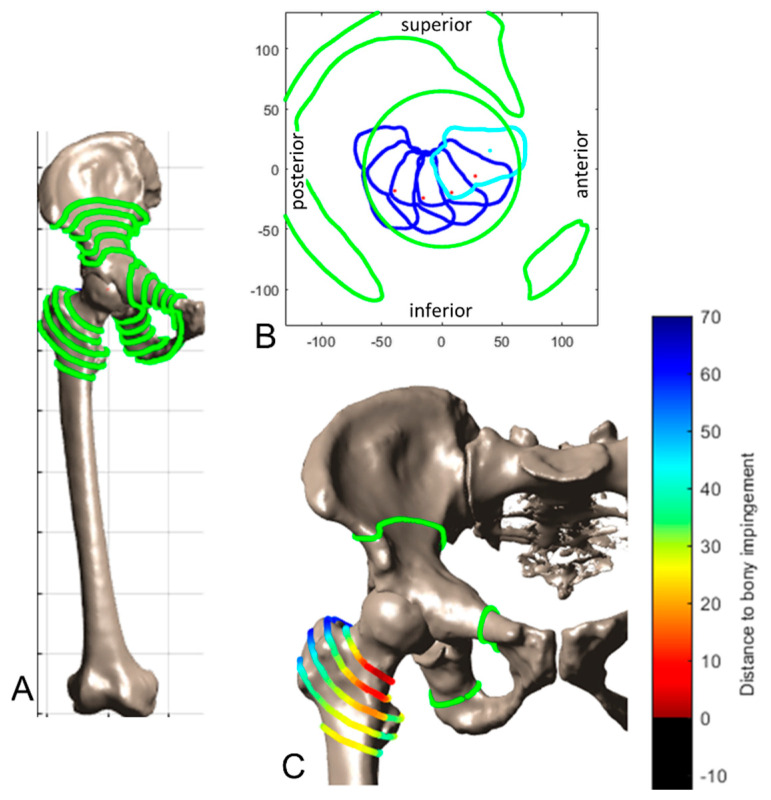
3D and 2D visualization of possible impinging points (PIP). (**A**): The PIPs are depicted in green on the femur and pelvis. (**B**): 2D mapping containing the cup and pelvic limits (green) for an exemplary flexion motion of the femur (blue). (**C**): The femoral PIP color-coded by the minimal distance to impingement.

A method introduced by our group is used for calculating the prosthetic ROM-based target zone [29]. Impingement is detected using a 3D to 2D mapping and a 2D distance map function and by evaluating the position of the neck axis relative to the cup limits, including the head/neck ratio. The pelvic tilt angle in standing position is applied to the pelvis before performing the motion of the femur defined by the target ROM relative to the pelvis [29]. In this study, the target ROM proposed by Sugano was selected defined by 120° flexion, 40° extension, 40° abduction and 40° internal rotation at 90° flexion [66]. Additionally, the supine pelvic tilt was considered in the calculation of the ROM-based safe zone, with a modified target ROM with 90° flexion, 5° extension, 30° internal rotation at 90° flexion and 30° external rotation at neutral flexion. For calculating bony impingement, our method was extended to incorporate arbitrary surface shapes. Potential impingement points (PIP) are derived by calculating the intersection of the femoral and pelvic surface with spheres of different radii positioned in the hip joint center. Figure 4A shows the PIP between the femur and the pelvis. Then, the mapping as described by Hsu is performed to calculate the minimal distance to impingement [29]. Figure 4B shows exemplarily the results of the mapping function for a flexion motion. Figure 4C shows the resulting minimal distances for each PIP. Instead of evaluating the absolute distance, the decrease compared to the preoperative situation is calculated for the bony ROM.

All implant and implantation parameters that satisfy the following criteria are considered as within the ROM-based target zone:

- No prosthetic impingement: distance to prosthetic impingement greater than 0°.
- A decrease of the bony ROM of less than 5° compared to the preoperative situation.

These thresholds can be adjusted to the individual patient or other standard values. Due to a slight medialization of the rotation center, most patients had a slight decrease of the bony ROM. Therefore, a small threshold for bony ROM decrease of 5° was chosen arbitrarily. 

#### 2.1.3. Load-Based Target Zone

**Figure 5:**
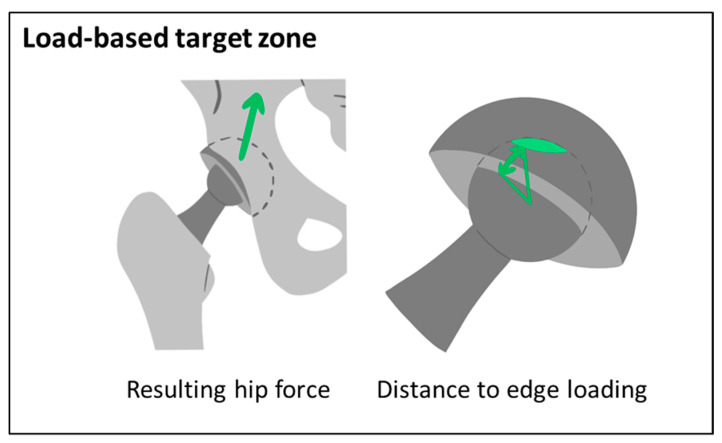
Criteria considered for the load-based target zone.

The amplitude and orientation of the resulting hip force and the resulting minimal distance to edge loading are calculated for the load-based target zone and compared to the preoperative situation (Figure 5).

The resulting hip force in one-leg stance as a surrogate for the peak force phase of level walking is calculated. Firstly, a cadaver template is patient-specifically adapted. The TLEM2.0 cadaver is individually scaled by deforming the femur and pelvis based on bony landmarks of the patient’s preoperative data, as well as the bony and prosthetic landmarks of the postoperative data. Then the pelvis of the scaled template is aligned by the patient-specific standing pelvic tilt. The scaled and aligned template serves as input to calculate the hip force using an approach proposed by our group [67]. Then, the contact patch between the femoral head and the cup is calculated using a model described by Imado et al. [68]. The distance to edge loading is defined as the minimal angular distance of the boundary of the contact patch to the rim of the cup. This was calculated using the same method as for calculating the distance to impingement, as described above and in [29].

All implant and implantation parameters that satisfy the following criteria are considered as within the load-based target zone:

- No edge loading: a minimal distance to edge loading greater than 0°.
- No increase of the resulting hip force: a decrease of the resulting hip force compared to the preoperative situation.

These thresholds can be adjusted to the individual patient or other standard values.

### 2.2. Retrospective Analysis

**Figure 6:**
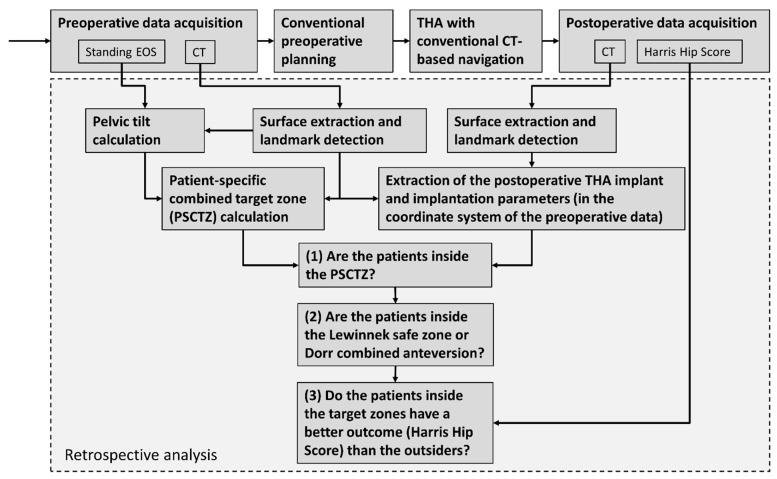
Overview of the retrospective study design.

Figure 6 gives an overview of the study design. All patients were operated using conventional preoperative planning and CT-based navigation by one surgeon (KT). For the retrospective analysis, the anatomical and functional data needed for the PSCTZ calculation were extracted from the preoperative data. The actual implant and implantation parameters were extracted from the postoperative data. Whether the patients were inside the PSCTZ, the Lewinnek safe zone and combined anteversion, and whether the patients inside the target zones have a better outcome, were analyzed.

The data of 201 THA patients was retrospectively selected to apply the method described above. There were 171 female and 30 male patients with a mean age of 62.9 years (range 34 to 91 years), a mean height of 1.56 m (range 1.40 to 1.82 m), a mean weight of 57.1 kg (range 35.9 to 103.8 kg), resulting in a mean BMI of 23.4 kg/m^2^ (range 16.6 to 34.5 kg/m^2^).

The diagnoses before surgery were osteoarthritis (183 patients), idiopathic osteonecrosis of the femoral head (six patients), subchondral insufficient fracture of the femoral head (six patients), femoral acetabular impingement (five patients) and acetabular fracture (one patient). All THA were performed using an anterolateral modified Watson-Jones approach in the lateral position. The CT-based planning and navigation systems used were LEXI ZedHip, Brainlab VectorVision Hip 3.5 or Stryker Hip Navigation. Implanted cups include Zimmer Continuum, Zimmer G7 OsseoTi, Kyocera SQRUM and Stryker Trident. The stems used were CLS, Modulus, Kyocera J-Taper HO, Stryker Accolade and Stryker Accolade II. Pre- and postoperative supine CT images with an isometric pixel spacing of 0.76 mm and a slice thickness and distance of 1 mm of the entire pelvis and both femurs and standing EOS images of the lower extremities including the entire pelvis were acquired from each patient. One patient was eliminated from the study due to missing slices in the postoperative CT images. The postoperative Harris Hip Score (HHS) [69] after one year was available for 199 patients. Therefore, the cohort consisted of 198 patients.

The preoperative CT images were semi-automatically segmented. The thresholds for bone were set to 200 and 2000 Hounsfield units. The surfaces were processed as described in a previous paper of our group [70]. The resulting meshes had a minimum and maximum edge length of 0.5 and 100 mm and a maximum deviation of 0.05 mm compared to the CT segmentation. The surface data served as the input for the PSCTZ calculation. The pelvic landmarks and coordinate system were automatically identified using the ITP method [70]. The landmarks required for the calculation of the femoral coordinate system were detected using the A&A method [71]. The automatically detected landmarks were reviewed and additional landmarks were manually identified by one experienced expert. To measure the preoperative standing pelvic tilt, the segmented surface of the pelvis was registered to the biplanar EOS images using the CT2EOS method [57].

The bony surfaces of the postoperative CT images were reconstructed similar to the preoperative data. Areas with strong artifacts induced by the implant were omitted. The implants were segmented using a threshold of 2000 Hounsfield units. In case of a ceramic inlay, the cup and the head were manually separated by using a sphere. Spheres and circles were fitted to the reconstructed surfaces of the cup and stem to derive the center of rotation, neck axis, neck, head and outer cup radius and cup orientation. The cup orientation is calculated according to Murray’s radiographic definition [72] relative to the pelvic coordinate system. Two landmarks were manually selected on the proximal and distal end of the surface model of the stem to define the stem axis (similar to [73]). The neck and stem axis were used to calculate the stem orientation, stem position, CCD angle and neck length using homogenous matrix operations [74]. The postoperative were registered to the preoperative bone models to describe the implant alignment relatively [57].

Subsequently, it was evaluated which patients were inside the PSCTZ, Lewinnek safe zone and Dorr combined anteversion range. The HHS was used for a comparison of the outcome of the patients inside and outside the target and safe zones. The HHS is not normally distributed. Therefore, two-sided Wilcoxon rank sum test was performed to test the difference. The statistical significance level was set at α = 0.05. It was also evaluated whether the age was similar between the insiders and outsiders using the same method.

## 3. Results

Complications after THA were the followings: two dislocations, one impingement, five psoas syndromes, eight greater trochanter fractures, one acetabular fracture, two peroneal nerve palsies, one sciatic nerve palsy, one stem subsidence and one ectopic ossification. The number of patients inside the morphology-based, ROM-based, load-based and inside all three single target zones and, therefore, inside the PSCTZ are listed in Table 1. The calculated values of each criterion are presented in Table 2.

**Figure 7:**
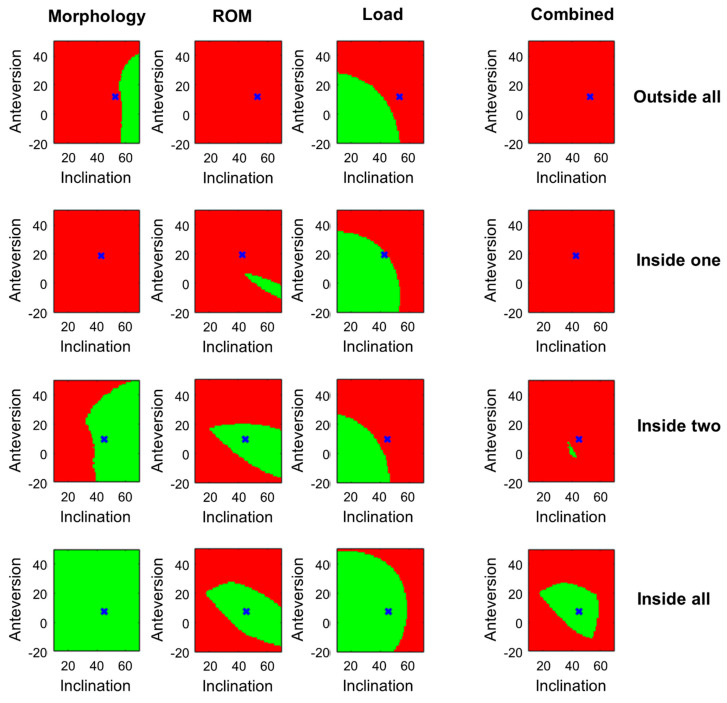
Patient-specific morphology-based, ROM-based, load-based and combined target zone for four exemplary cases. The target zones for the implant and implantation parameters based on the postoperative CT data is shown. The blue x marks the measured postoperative cup orientation.

Figure 7 shows four exemplary cases which are inside none, one, two or all three patient-specific target zones.

**Figure 8:**
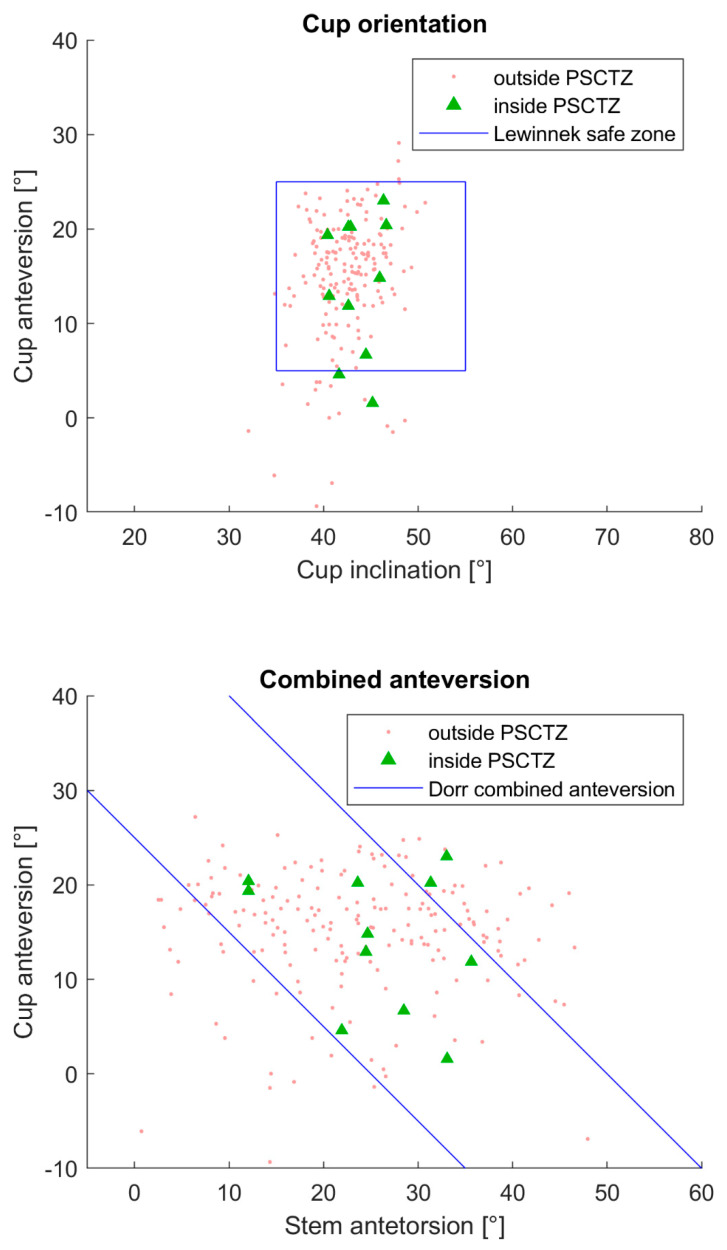
Distribution of the cup orientation and the combined anteversion of the PCSTZ-insiders and outsiders. The conventional safe zones by Lewinnek and Dorr are also visualized.

The distribution of the cup orientation and combined anteversion of the patients inside and outside the PSCTZ and the range of the conventional safe zones are shown in Figure 8. Two PSCTZ insiders were outside the Lewinnek safe zone and two outside the Dorr combined anteversion range.

**Figure 9:**
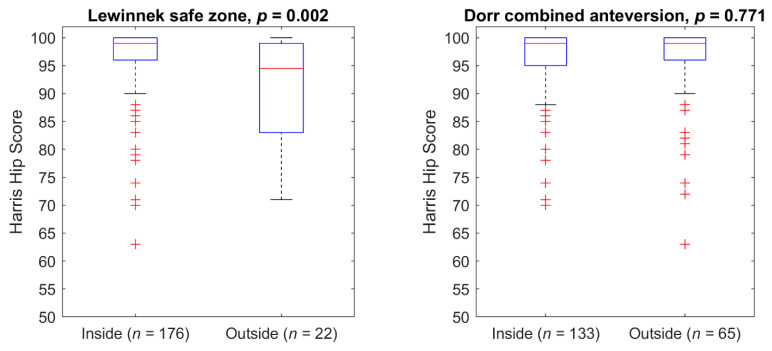
The HHS of the patients divided by the conventional target zones.

The HHS of the patients inside and outside the conventional safe zones are shown in Figure 9 and Table 1. Patients inside the Lewinnek safe zone had a significantly higher HHS and are also significantly younger compared to the outsiders. No significant difference existed regarding the HHS and age between insiders and outsiders for the Dorr combined anteversion range. 

**Figure 10:**
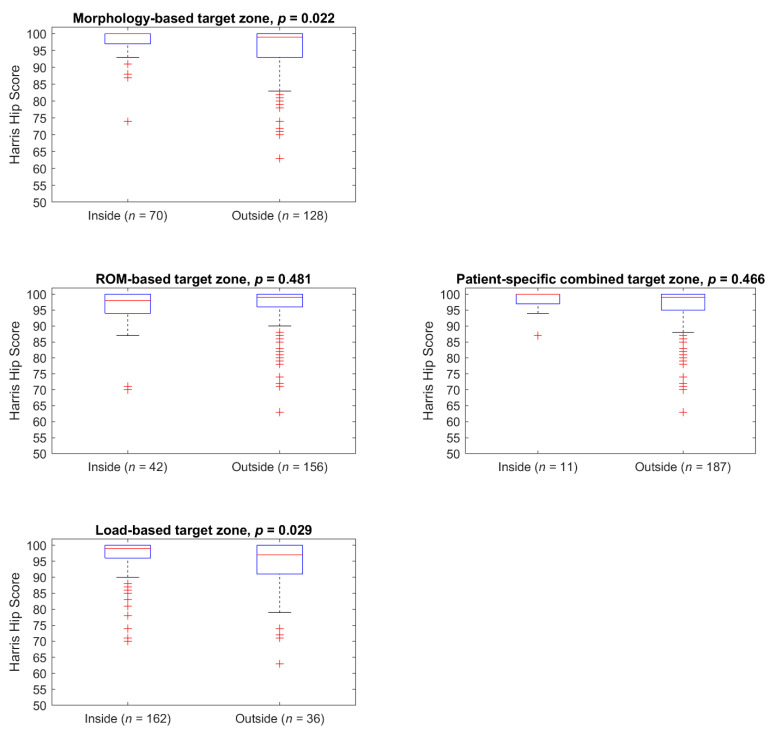
HHS of the patients divided by individual and combined target zones.

Figure 10 and Table 1 show the HHS of the patients inside and outside the individual target zones. Insiders of the morphology-based target zone had a significantly higher score than the outsiders. No significant difference in age was found. No significant difference was found between insiders and outsiders of the ROM-based target zone regarding HHS and age. For the load-based target zone, insiders had a significant higher median HHS and a significant younger age compared to the outsiders. No significant difference between the two groups was evident for the PSCTZ. In all single target zones, some insiders have low HHS. Only if the target zones were combined into the PSCTZ were almost all patients with a low HHS removed. 

## 4. Discussion

A method for calculating suitable THA implant and implantation parameters for a specific patient considering the most relevant criteria mentioned in the literature was introduced. Additional criteria can be added in a modular fashion. The majority of patients in the cohort show an excellent outcome based on the high median HHS. Fewer patients with an HHS below 95 can be observed inside the PSCTZ. If patients are divided by the individual target zones, the difference is less obvious. Only when all target zones are combined were most outliers with lower scores removed. This stresses the importance of combining all relevant criteria into the planning process. It should be further analyzed how each target zone contributes to the PSCTZ. Only 6% of patients were inside the PSCTZ. More patients inside the PSCTZ might be necessary to detect a significant difference between insiders and outsiders. This could be achieved by a larger cohort or prospective study. 

A prospective study would also enable a division of the patients into a treatment and a control group with an even sampling of the cases regarding age, comorbidities and other factors which might affect the postoperative outcome. The current results show that, for some groups, a significant higher HHS coincides with a significantly younger age. 

The number of patients inside the Lewinnek safe zone and the Dorr combined anteversion range is very large compared to the number of patients inside the PSCTZ. One reason for the latter might be that not all the criteria used for the PSCTZ were considered during the planning and implantation process, since the commercial planning systems could not offer the method proposed in this paper. Figure 9 and Figure 10 show that the PSCTZ is more conservative than the conventional safe zones. It provides a refined target for the cup orientation and does not violate the recommendation specified by Lewinnek for most of the patients. In the current study, 167 patients were inside the Lewinnek safe zone but outside the PSCTZ. Based on a cohort of 206 patients with dislocations after THA, Abdel et al. showed that 58% of them were within the Lewinnek safe zone [10]. It would be interesting to know whether these patients were inside the PSCTZ. For a related detailed analysis, 3D data of the bony anatomy and implant and implantation parameters of the patients would be required.

Whether the HHS is an adequate score for measuring the clinical outcome of THA is questionable [75]. The HHS reflects certain aspects such as pain and the ability to perform some ADLs. The ROM and anatomical deformities are considered, but have a minor impact on the overall score. Whether a patient had a dislocation is not represented by the score. Mid- and long-term aseptic loosening and wear can be related to resulting hip forces and edge loading, but might be not reflected in a short-term HHS evaluation. Hence, the HHS may not represent all criteria used for PSCTZ calculation. A significant difference in HHS is not necessarily clinically significant. In addition to the HHS, other outcome measures quantifying the quality of life of the patient, such as the Western Ontario and McMaster Universities Osteoarthritis Index (WOMAC) or hip disability and osteoarthritis outcome score (HOOS) might be considered in the future. However, these outcome measures might exhibit the same limitations as mentioned for the HHS.

The cohort contains only Japanese patients. Data of patients from other countries are needed for a further validation of the PSCTZ. The resulting PSCTZ strongly depends on the definition of target ROM and the thresholds of minimal bony coverage, residual bone thickness, maximum anterior cup overhang, distances to impingement and edge loading, and decrease in the resulting hip force. Whether the thresholds are chosen adequately in this study has to be further evaluated. In the future, the thresholds could be defined or adapted according to patient-specific requirements.

The standing pelvic tilt might change after THA [76,77], which changes the functional cup orientation. Using the preoperative pelvic tilt might not reflect the postoperative situation. Especially if the cup orientation is already at the boundary of the PSCTZ, a different pelvic tilt might cause the implant to be outside of the PSCTZ. Therefore, a prediction of the postoperative pelvic tilt from preoperative data as proposed in a recent study of our group could be useful [57]. Other studies recommend the inclusion of additional pelvic tilts, such as sitting, in the ROM and load analysis [47]. Unfortunately, we could only include standing and supine pelvic in the ROM analysis, hence other pelvic tilts were not available for the cohort.

The ROM-based target zone considers only prosthetic and bony impingement. Other planning software also examines bone to implant impingement [78,79]. The approach to detect bony impingent introduced in this study should be compared with other approaches for impingement detection, for instance a collision detection algorithm. The latter also allows for integration of asymmetric cup designs, which is not possible with the prosthetic impingement method used in this study. Osteophytes were not removed from the preoperative pelvic surface models, which might lead to false bony impingement detection in case the osteophytes are resected during THA. These might be reasons for the missing significant difference between in- and outsiders of the ROM-based target zone.

Additional limitations of the study have to be considered. The calculation of the leg length was based on the knee joint, since CT data of the ankle joint was not recorded. The leg length discrepancy might be incorrect if it results from a difference of the lower limbs. 

Although different prediction models for the hip force are already commercially used in 2D and 3D preoperative planning of THA [41,80], the validity of the hip force prediction and the contact patch calculation for different bearing surfaces has to be further evaluated [67].

## 5. Conclusions

Numerous criteria are relevant for THA planning, but conventional planning methods do not systematically consider these together in an integrated approach. The proposed method calculates a PSCTZ including the most relevant criteria. More criteria could and should be added into the modular framework. A retrospective analysis shows that, for a prosthesis implanted in the PSCTZ, a low outcome score of the patient is less likely than using the conventional safe zones. This finding should be further evaluated in a prospective study.

## Figures and Tables

**Table 1:** Number of patients inside and outside the conventional and patient-specific target zones and their median HHS and age.

	Inside				Outside				
	*n*		Median (Min., Max.)	Percentage of Insiders below a HHS of 95 (%)	*n*		Median (Min., Max.)	Percentage of Insiders below a HHS of 95 (%)	*p*
**Lewinnek safe zone**	176	HHS	99 (63, 100)	20	22	HHS	95 (71, 100)	50	0.002
		Age	62 (34, 91)			Age	70 (54, 85)		0.000
**Dorr combined anteversion**	133	HHS	99 (70, 100)	24	65	HHS	99 (63, 100)	23	0.771
		Age	63 (34, 87)			Age	62 (38, 91)		0.600
**Patient-specific target zones**									
**Morphology-based**	70	HHS	100 (74, 100)	14	128	HHS	99 (63, 100)	29	0.022
		Age	62 (38, 87)			Age	63 (34, 91)		0.256
**ROM-based**	42	HHS	98 (70, 100)	26	156	HHS	99 (63, 100)	23	0.481
		Age	67 (51, 82)			Age	63 (34, 91)		0.110
**Load-based**	162	HHS	99 (70, 100)	20	36	HHS	97 (63, 100)	39	0.029
		Age	62 (34, 87)			Age	70 (38, 91)		0.008
**PSCTZ**	11	HHS	100 (87, 100)	18	187	HHS	99 (63, 100)	24	0.466
		Age	62 (52, 82)			Age	63 (34, 91)		0.972

**Table 2:** Calculated values of each criterion of the PSCTZ.

Criterion	Median (Q1 to Q3, Min. to Max.)	Mean ± SD
**Cup coverage (%)**	79.6 (72.0 to 86.6, 49.8 to 97.7)	78.7 ± 9.8
**Max. Anterior cup overhang (mm)**	9.1 (4.4 to 14.8, 0.0 to 38.5)	9.9 ± 7.2
**Distance prior to cup penetration (mm)**	2.0 (0.4 to 3.7, −4.5 to 9.1)	2.0 ± 2.6
**Leg length discrepancy (mm)**	0.8 (−3.8 to 5.2, −48.1 to 27.0)	0.8 ± 8.4
**Distance to prosthetic impingement (°)**	−0.4 (−4.1 to 2.2, −17.1 to 10.6)	−1.1 ± 5.1
**Decrease of bony ROM (°)**	0.6 (−4.6 to 6.3, −32.7 to 18.3)	−0.2 ± 8.9
**Min. distance to edge loading (°)**	5.6 (1.6 to 8.7, −14.9 to 21.6)	5.1 ± 6.3
**Decrease of the resulting hip force (%BW)**	−0.6 (−1.0 to −0.3, −8.7 to 2.4)	−0.7 ± 0.9

## Data Availability

The datasets generated during and/or analyzed in this study are available from the corresponding author upon reasonable requests.
